# Ultrasound-assisted pseudohomogeneous tungstate catalyst for selective oxidation of alcohols to aldehydes

**DOI:** 10.1038/s41598-022-06874-5

**Published:** 2022-03-01

**Authors:** Aram Rezaei, Yasaman Mohammadi, Ali Ramazani, Huajun Zheng

**Affiliations:** 1 Nano Drug Delivery Research Center, Health Technology Institute, Kermanshah University of Medical Sciences, Kermanshah, Iran; 2 Department of Chemistry, University of Zanjan, Zanjan, Iran; 3 Department of Applied Chemistry, Zhejiang University of Technology, Hangzhou, 310032 China

**Keywords:** Green chemistry, Materials chemistry, Organic chemistry, Surface chemistry, Chemical synthesis

## Abstract

The idea of applying ultrasound (US) as a green activation method in chemical transformations, especially in catalytic alcohol oxidations, technically and ecologically appeals to chemists. In the present work, as an attempt to fulfill the idea of designing an eco-friendly system to oxidize alcoholic substrates into corresponding aldehydes, we developed multifunctional tungstate-decorated CQD base catalyst, A-CQDs/W, and examined its sonooxidation performance in presence of H_2_O_2_ as a green oxidant in aqua media. By comparing the catalyst performance in oxidize benzyl alcohol as a testing model to benzaldehyde (BeOH) prior and after US irradiation—trace vs 93%- the key role of ultrasonic irradiation in achieving high yield is completely appreciated. Exceptional thermal and compression condition that is created as a result of acoustic waves is in charge of unparalleled yield results in this type of activation method. The immense degree of reagent interaction in this method, ensures the maximum yield in notably low time, which in turn leads to decrease in the number of unreacted reagents and by-products. Meanwhile, the need for using toxic organic solvents and hazardous oxidants, auxiliaries and phase transfer catalyst (PTC) is completely obviated.

## Introduction

The high demand for various types of aldehydes as an essential intermediate to produce broad range of materials, such as toiletries, perfumes, pesticides and dyes, in addition with using a great deal of this substrates in pharmaceuticals and agribusiness industries, justifies the huge number of studies and great deal of attention that is dedicating to this field. Catalytic alcohol oxidation is a popular method to synthesize aldehydes, but despite the favorable outcome in many cases, establishing an eco-friendly method to producing these intermediates in high yield and selectivity still remain as a main challenge. In order to take a number of green chemistry criteria into practice, the amount of harmful and toxic solvents and reagents should be kept at the lowest level or be substituted with non-toxic and benign materials, additionally, the amount of energy consumption in reactions should be diminished^1^. It seems no exaggeration to say that among all of the new green activation methods up until now namely; Photochemistry^2,3^, Microwave^4^, Biotransformation^5,6^, and Ultrasound, no technique accommodates green chemistry principles in chemical transformations as ultrasound does. Besides the widespread applications of ultrasound in industry, medical section and navigation, chemistry and chemical syntheses have been taking advantage of this effect too. One of the well-known applications of ultrasound in chemistry is associated with degradation of water contaminants such as azo dyes^7,8^ but there are plenty of examples in which ultrasound is used as a green activation energy to synthesize various chemicals^9,10^ in this way not only the need for applying external heating to proceed the reactions is eliminated, but also the amount of catalyst, dangerous solvents, polluting PTC and axillaries is cut down. Physical effects arising from ultrasound waves can cause important chemical changes at the molecular level, but according to the fact that the size of ultrasound waves that are nearly a few micrometers, are not consistent with the dimension of molecules, they could not be able to directly trigger a chemical change in molecules, thus, another. Phenomena would be in charge of this alteration- the concept that is referred to as cavitation^11^. Simply stated, as a result of applying acoustic ultrasound waves into reaction vessel, periodic impaction-expansion that is created in liquid along the direction of waves diffusion, could causes negative pressure in some parts of liquid which consequently leads to formation of micro cavities or vapor-filled bubbles that sometimes tend to grow ten times than that of their primary sizes. Extreme conditions inside the overgrown bubbles because of the existence of huge deal of energy which is trapped inside them could lead to bubble explosion and ultimately in a split second and localized area, extraordinary amount of energy in terms of heat and pressure releases into the liquid- exceed the 5000°k and 1000 bar^11^. Agitation that is created as a result of these shockwaves is capable of enhancing the mass transfer in far higher extent compared to that of conventional magnetic stirring. So, as a result of remarkable reagent interaction, exceptional heat and pressure, reactions could proceed kinetically at a faster rate than that of traditional thermally activated reactions. In this way, high yields would be achieved in the lowest reaction time and least dosage of catalyst, meanwhile the amount of waste and byproducts could be kept in minimum level. However, despite all the advantages of using ultrasound for chemical syntheses, there are only few reports in which ultrasound is used for alcohol oxidation reactions. Mahamuni et al. utilized the ultrasound for catalytic oxidation of benzyl alcohol to BeOH by (dodeca tungsto phosphoric acid) as a homogeneous catalyst in presence of (Aliquat-336) as PTC, hydrogen peroxide as oxidant and methylene chloride as solvent^12^. During a 135 min reaction time, they observed that there was a remarkable difference between the amount of BeOH that was produced in presence of ultrasound irradiation compared to silent condition. However, in spite of yield improvement that was achieved in this system, environmental harms that are inflicted because of applying toxic solvent or adding PTC into reaction vessels, in addition, difficulties associated with separating homogeneous catalyst afterward, are some of the downsides with this work. In another work Chevallier et al. studied the benzyl alcohol transformation into BeOH in presence of a whole range of metal oxide, metal nanoparticles and metal salts as catalyst species in aqua media by using H_2_O_2_ as oxidant^13^. Similarly, the best results obtained in the presence of ultrasound waves compared to the ultrasound-free condition, (26.2 vs 19.6%). Although the total yield is low, we can conclude the key role of ultrasound irradiation in achieving enhanced yield. To the best of our knowledge there are few reports on using carbon base catalysts in sonocatalytic reactions, still less, no report on ultrasound assisted oxidation of benzyl alcohol or its derivatives by carbon quantum dot-based catalysts^14,15^. Carbon quantum dots (CQDs) are a new emerging class of carbon-based nanomaterials that because of their unique characteristics such as high stability, good conductivity, high solubility in aqua media, in addition, biocompatibility, nontoxicity and ease of synthesis, are widely used in various fields such as, bioimaging, biosensor industry and drug delivery. Furthermore, excellent luminescence, optical and electrical properties associated with CQDs, render them as a suitable option in biomedicine, optoelectronic and catalysis fields^16,17^. Besides, small size of these particles, less than 10 nm, and abundant oxygen-rich surface functional groups such as hydroxyl and carboxyl, caused them to be a proper option for loading a wide range of organic and inorganic modifiers, active species and nanoparticles on its surface, which is a highly precious feature when it comes to choose a proper support for a catalyst. Tungstate anions WO_4_^=^ as a powerful activation agent for oxidation of alcohols or epoxidation of alkenes into corresponding aldehydes or ketones are utilizing widely in catalytic alcohol oxidation practices^18–20^. Although proper stabilization of these species on catalyst support in a way metal etching be kept at a minimum level still remains as a big challenge. The possibility of grafting a broad range of organic and inorganic modifiers on CQDs presents them as the suitable platforms to properly host diverse kinds of active sites such as tungstate species^21^.

Moreover, in terms of total catalyst costs, when calculating there are two main factors that should be taken into consideration. First the metal type and second energy consumption. In terms of metal type, this catalyst could be regarded as one of the most low-cost catalysts as tungstate is a inexpensive metal with invaluable features such as great catalytic activity, non-toxicity, availability, good oxygen carriage ability, and great capability towards activating H_2_O_2_ which makes it an appealing item for oxidation purposes. The reason why tungstate base catalysts sometimes are considered as pricey catalysts has something to do with the wrong choice of support and the methods through which tungstate is loaded on support^18–20,22,23^.

There are numerous reports on W-H_2_O_2_ systems which each tried in some way immobilize tungstate on the support, such as using polymers, magnetic nanoparticles or mesoporous silica. But these kinds of supports suffer from the high metal etching during the reaction that obligate the need for loading an excess amount of tungstate to offset the metal etching during reaction and in this way keep the reaction yield in a good level which have proved to result in high-priced catalyst. In this work by adopting CQDs, and subtle choice of modifiers such as ionic liquids (ILs), we could overcome all these challenges effectively (Table 1)^24–30^.

**Table 1:** Comparative table of reported methods for the oxidation of benzyl alcohol.

	Catalyst	Condition	Conversion (%)	Selectivity (%)	References
1	[Imidazolium]_3_[PO_4_(W(O)(O_2_)_2_)_4_]^3−^	Catalyst (0.05 mmol), [bmim][BF_4_], H_2_O_2_: BeOH (2:1), 90 °C, 8h	–	78	^24^
2	PW/DAIL/MIL-101(Cr)	Catalyst (6 μmol), TBHP: BeOH (4.5:1), CHCl_3_,100 °C, 6h	95	99	^25^
3	WO_4_^=^@PMO-IL	Catalyst (1.5 mol%), H_2_O_2_: BeOH (5:1), MeCN: H_2_O (1:1),(50 mmol), 90 °C, 12 h	75	100	^26^
4	CoFeO_4_ (Ultrasound)	Catalyst (1 mol%), H_2_O_2_: BeOH (1:1), H_2_O, 70 °C, 15 min	16.7	1.6	^27^
5	FeCl_3_/HNO_3_ (Ultrasound)	Catalyst (0.5 mmol), BeOH (1 mmol), acetone, r.t., 10 min	–	94	^28^
6	CuSO_4_.5H_2_O (Ultrasound)	Catalyst:KMnO_4_ (1:1, 2.2 g), BeOH (1 mmol), CH_2_Cl_2_, 30 min	73	> 97	^29^
7	GO (Ultrasound)	Catalyst (200 wt%), BeOH (1 mmol), toluene, 80 °C, 2h	–	98	^30^
8	A-CQDs/W (Ultrasound)	Catalyst (1 mol%), H_2_O_2_: BeOH (3:1), r.t., 3 min	98	93	This work

Herein, we developed amphiphilic multifunctional CQDs base catalyst, A-CQDs/W to oxidize a range of alcoholic substrates into corresponding aldehydes. Specific design of catalyst by grafting both hydrophilic and hydrophobic groups on the surface of CQDs such as [APMIm][Cl] and stearic acid, not only caused to effectively immobilize active moieties of WO_4_^=^ on CQDs and prevent any metal ion erosions, but also facilitates conducting the reaction in benign aqua media and ensures the maximum efficient connection between alcoholic substrate and active sites of catalyst. On the other hand, by introducing ultrasound waves, reactions could readily carry out in ambient temperature and pressure, thus the need for reflux setup and spending high time and energy to complete the oxidation reaction is obviated. In this way a number of green chemistry principles such as; prevention, atom economy, less hazardous chemical synthesis, designing safer chemicals, safer solvents and auxiliaries, energy efficacy, reduced derivatives, and operator safety could come true^31^. At the end, in order to characterize the structure of the prepared catalyst, FT-IR, XRD, TGA, FESEM, TEM, NMR, UV–vis, PL and EDS analyses were performed.

## Experimental section

### Materials and methods

All reagents and chemicals were purchased either from Fluka company (Switzerland) or Merck company (Germany). Both A-CQDs and A-CQDs/W samples were characterized by following methods; Fourier-transform infrared (FT-IR) spectroscopy of samples recorded using KBr pellets which were performed by PerkinElmer PE-1600-FTIR spectrometer. X-ray diffraction (XRD) patterns obtained using X′PretPro diffractometer, Panalytical-Holland, with Cu Kα radiation (λ = 1.54 A). Thermogravimetric analysis (TGA) performed by a TGA Q 600 analyzer, TA-America, under Ar flow at a heating rate of 20 ºCmin^−1^. UV–vis’s spectrophotometry of samples measured using Shimadzu UV 2100 151PC UV–Visible spectrophotometer at room temperature. Field emission scanning electron microscope (FE-SEM) imaging was carried out using a MIRA III, TESCAN-Czech Republic. Transmission electron microscopy (TEM) imaging technique was performed by an EM 208S electron microscope and conducted at 100 kV. energy-dispersive X-ray spectroscopy (EDX) analysis was carried out on a SIGMA VP 500 (Zeiss) microscope equipped with an EDX measurement system. 1HNMR and 13CNMR evaluations were carried out with a BRUKER DRX-250 AVANCE spectrometer at 250.0 MHz and 62.5 MHz.

### Synthesis of A-CQDs

A-CQDs and A-CQDs/W were prepared as reported in our previous works^32^, in a typical hydrothermal method, Citric Acid (3 mmol), [APMIm][Cl] (4.5 mmol), Stearic Acid (1 mmol) and 1,2-Bis(3-aminopropylamino) ethane (3 mmol) were added to a solution contain of (1:2) ratio of (H_2_O: EtOH) in a round bottom balloon and sonicated for 5 min in ultrasonic bath. As obtained mixture transferred to a Teflon-lined stainless-steel autoclave of 25 ml and heated at 200 °C for 4 h under N_2_. The resulting mixture was allowed to cool to room temperature. Finally, viscose reddish-brown liquid refined via dialysis membrane (100 Da) and named A-CQDs.

### Synthesis of CQDs/W

The Na_2_WO_4_.2H_2_O (2 mmol) was dissolved in 2 mL denoised water in a round bottom flask, then certain amount of as prepared A-CQDs added to the aforementioned solution and allowed to stir overnight in ambient temperature. The obtained mixture was filtered by centrifugation (16,000 rpm) for 10 min. To further purification and remove unreacted substrates the filtrate purified via dialysis (100 Da) for 48 h and finally freeze dried 24 h. Resulting light brown powder denoted as A-CQDs/W.

### Sonocatalytic test

All sonocatalytic oxidation reactions of the synthesized catalyst were carried out in room temperature. Typically, 1 mmol BeOH and 1 mL deionized water were mixed in a round bottom flask next 1 mol % of as synthesized A-CQDs/W catalyst was added and stirred for 5 min, then ultrasound waves were applied in flask through placing ultrasound probe into flask, meanwhile, H_2_O_2_ (35% aqueous solution) was added to the solution slowly. After 3 min of ultrasound irradiation, in order to separate organic product from aqua media, flask content was transferred into separatory funnel and mixed with 2 mL DCM thoroughly to isolate organic phase.

## Results and discussion

### Catalyst characterization

**Figure 1:**
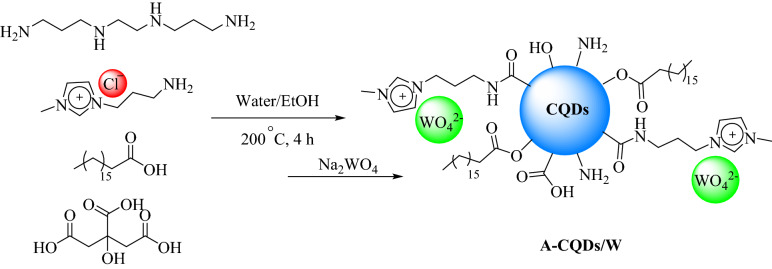
Schematic procedure for the synthesis of the A-CQDs/W catalyst.

The A-CQDs/W was synthesized through one pot hydrothermal process in 200 °C via combining certain amount of citric acid (CA) as carbon source, [APMIm][Cl] and 1,2-Bis(3-aminopropylamino) ethane as ion stabilizer and hydrophilic functional group and, Stearic Acid, as a hydrophobic group. In the second step, during the ion exchange process, via adding a certain amount of Na_2_WO_4_ to the water solution containing as prepared A-CQDs, chloride ions were exchanged with (WO_4_^=^) ones to give final, A-CQDs/W catalyst (Fig. 1).

**Figure 2:**
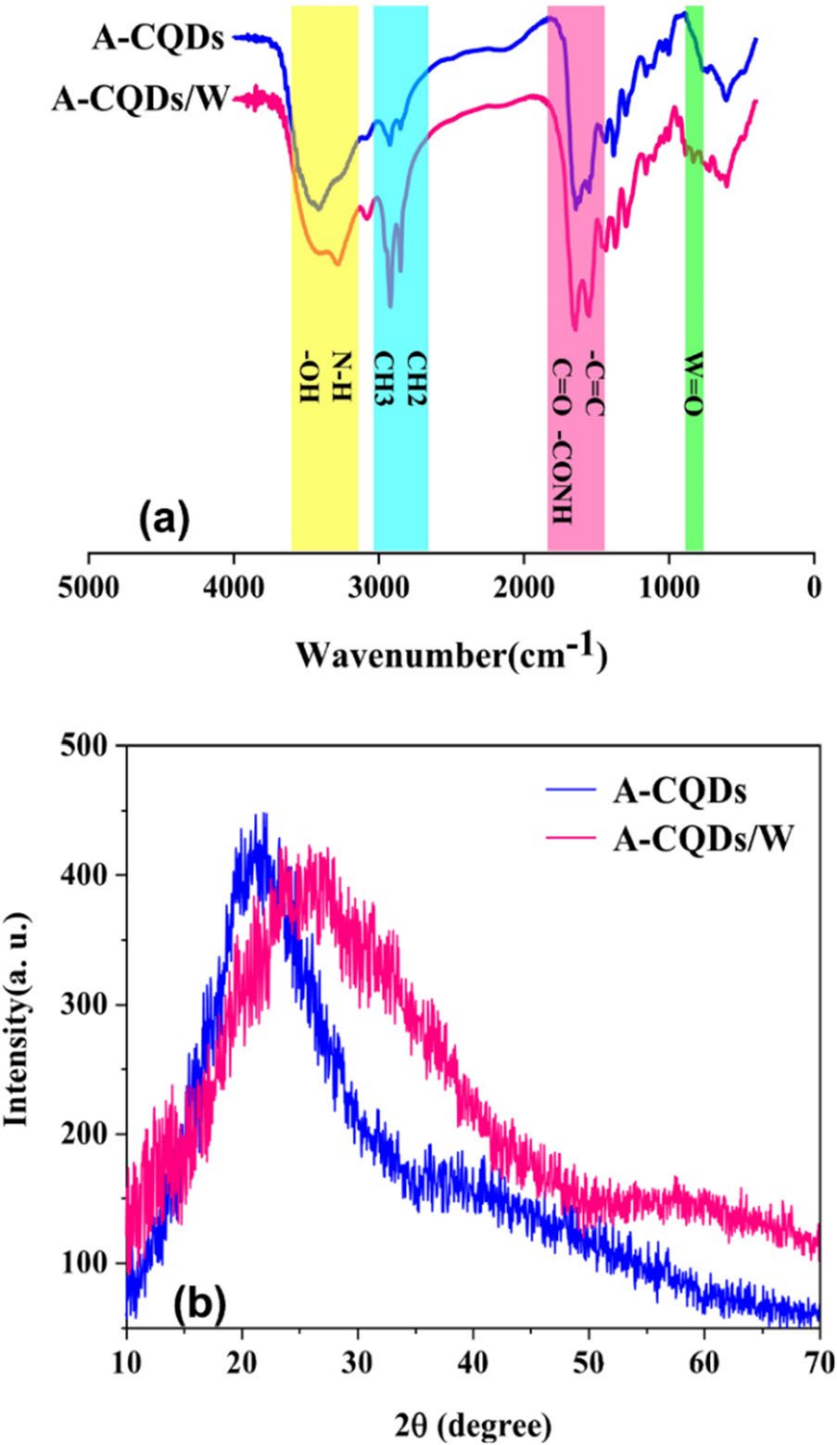
(**a**) FT-IR Spectra of A-CQDs (blue graph) and A-CQDs/W (pink graph), (**b**) XRD pattern of A-CQDs (blue graph) and A-CQDs/W (pink graph).

In order to identify the surface functional groups of A-CQDs and A-CQDs/W, FTIR analysis, was performed^23^. As can be seen in Fig. 2a, a well-established broad band at near 3418 cm^−1^ in both spectra, is related to O and N containing functional groups such as O–H and N–H stretching vibrations meanwhile bending vibrations of -OH caused a peak at 1382 cm^−1^. Asymmetric, symmetric, scissoring vibration modes related to -CH_2_ and -CH_3_ bands attributed to alkyl chains that originated from Stearic Acid and those of [APMIm][Cl] and 1,2-Bis(3-aminopropylamino) ethane are reflected at 2836, 2940, 1439 cm^−1^ respectively. Additionally, long-chain vibration modes belong to alkyl groups from Stearic Acid, are marked at about 730 cm^−1^. The amide carbonyl stretching mode and vibration of C=C groups appear at 1735 and 1565 cm^−1^, respectively. The two main characteristic peaks related to the imidazole ring, originating from [APMIm][Cl], involving C–N stretching vibrations, and -C-H bending modes, appeared at 1162 and 618 cm^−1^, respectively. Unlike the A-CQDs spectrum, presence of a light absorption peak at about 834 cm^−1^ attributed to stretching vibrations of W=O bond in A-CQDs/W sample, verifies exchange of tungstate ions with chloride ones over the ion exchange process. These data verify the successful synthesis of multi-functional CQDs with a variety of hydrophobic and hydrophilic groups grafted on it.

XRD graphs related to both samples displayed in Fig. 2b. As can be seen, in XRD patterns related to A-CQDs and A-CQDs/W, both samples show the typical broad peak related to amorphous carbonaceous compounds without any other distinct peak which confirms the formation of amorphous structure of CQDs core. As is shown characteristic peak corresponds to amorphous carbon network in A-CQDs displayed at about 21.25°, which after substitution tungstate ions with chlorine ones in A-CQDs/W, this peak slightly broadened and shifted toward higher angle and appeared at about 24.70^33–35^.

**Figure 3:**
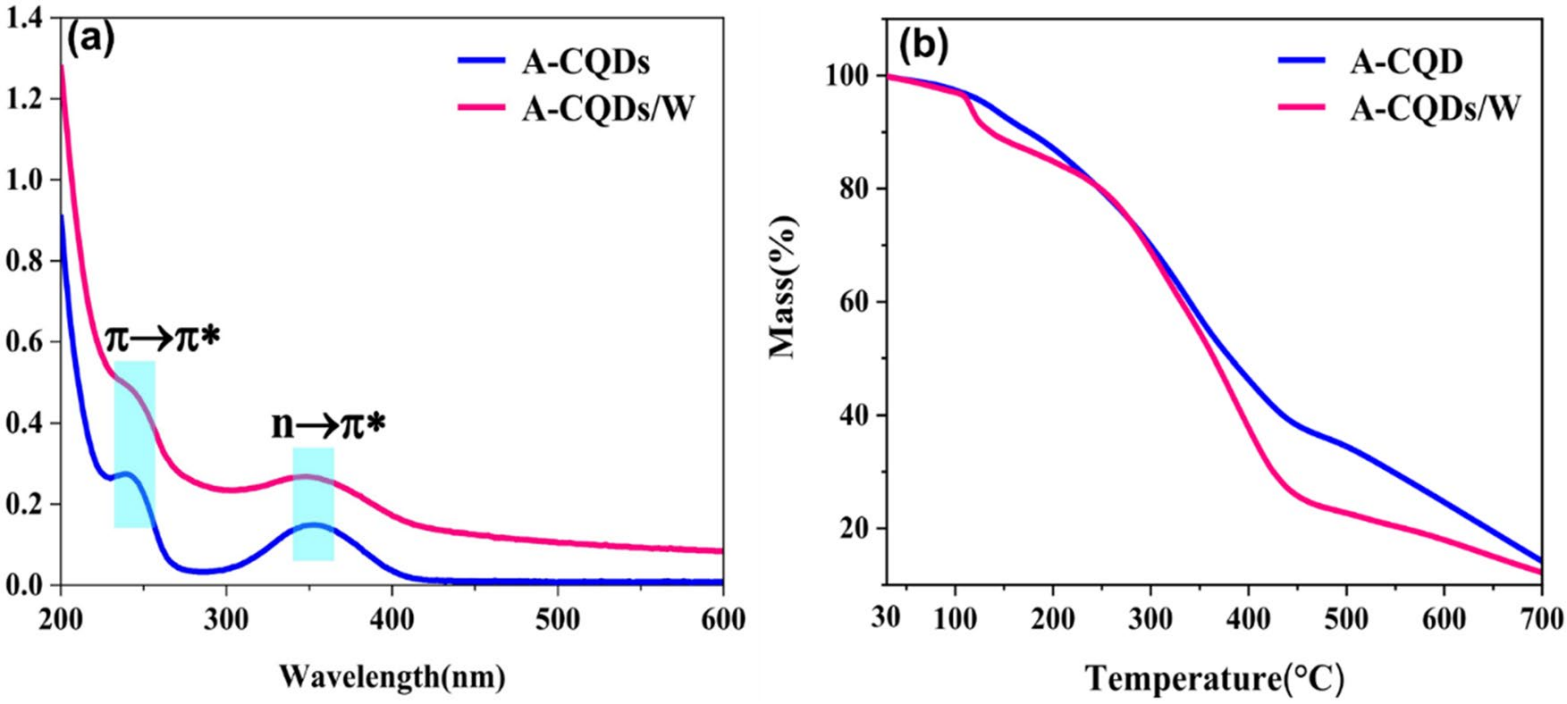
(**a**) UV–vis spectrum of A-CQDs (blue graph) and A-CQDs/W (pink graph), (**b**) TGA plot of A-CQDs (blue graph) and A-CQDs/W (pink graph).

UV–vis spectroscopy of as prepared A-CQDs and A-CQDs/W samples is shown in Fig. 3a. According to spectra, two characteristic peaks centered at 230 nm and 300–400 nm are attributed to π → π* and n → π* transitions, which are related to aromatic conjugated sp^2^ systems and non-bonding electrons respectively^23,36^. This evidence confirms the formation of CQDs core in both samples. Thermal gravimetric analysis (TGA) (Fig. 3b) was conducted in the range of 30–700 °C to investigate thermal stability and study the structure of samples. Totally, A-CQDs and A-CQDs/W samples experienced 86 and 88% weight loss through 3 minor and one major weight loss stages. Slight weight loss from 25 to 107 °C can be ascribed to desorption of remaining H_2_O or other solvent molecules trapped in catalyst structure that caused almost 3% weight loss in both samples alike. Second stage begins at around 110 °C and continues to 240 °C, at the end of this stage both samples experienced 19% decline in their weights similarly, however despite the gradual weight loss in A-CQDs sample in this stage, A-CQDs/W graph observed a drop at the first of this part at about 113 °C but then weight loosing was more gradual same as A-CQDs that at the end both graphs overlapped at 240 °C, generally reduction in this stage is attributed to disintegration of oxygen-containing functional groups such as carboxyl and carbonyl specious. Over the third stage, as temperature raised from 240 to 463 °C mass of samples fell considerably by 76 and 63% for A-CQDs/W And A-CQDs samples respectively that is attributed to decomposition of stearic acid, 1,2-bis(3-aminopropylamino) ethane and [APMIm][Cl] moieties loaded on CQDs surface^14,33^. Further increase in temperature caused moderate weight loss in samples that stemmed from degradation or graphene like structure of core CQDs. These data one more time verify the successful synthesis of multifunctional structure of catalyst with a variety of hydrophobic and hydrophilic specious grafted on its surface.

**Figure 4:**
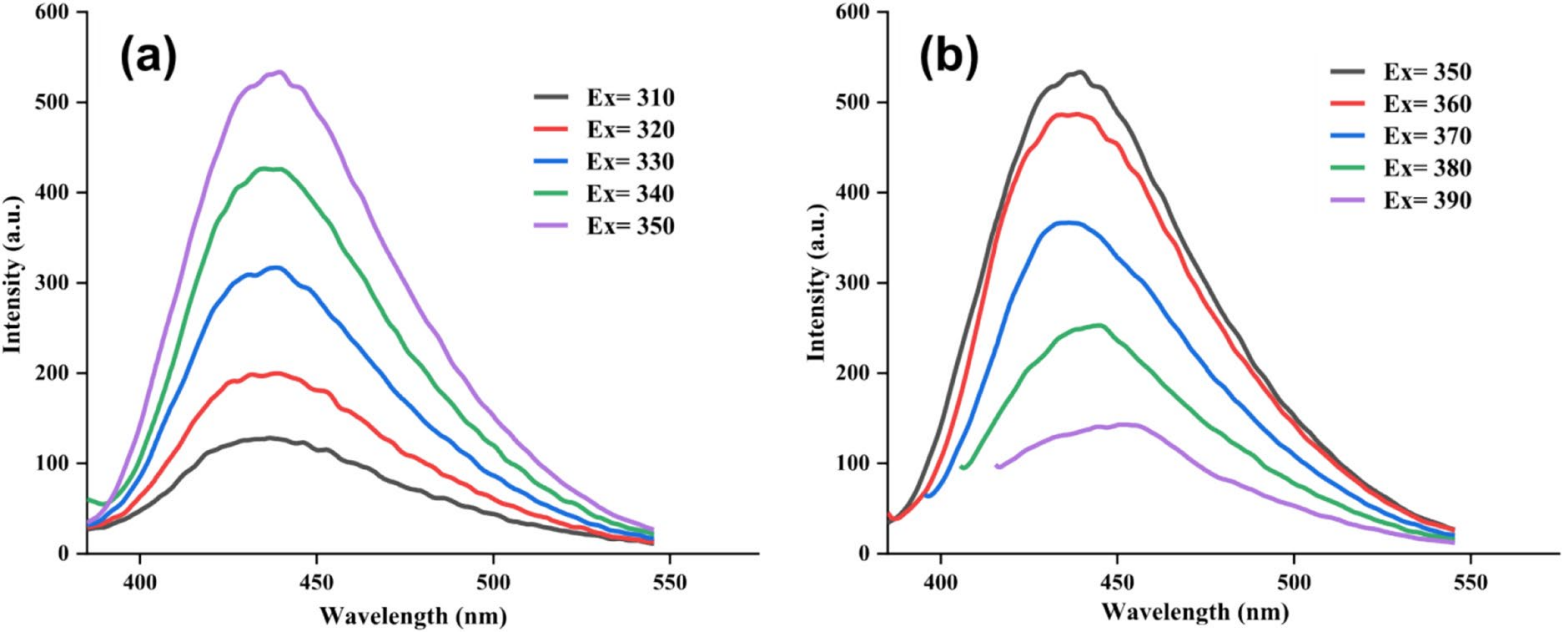
PL graphs of A-CQDs/W (**a**) Ex = 310–350 nm, (**b**) Ex = 350–390 nm.

Data from photoluminescence (PL) analysis related to A-CQDs/W and A-CQDs are presented in Figs. 4 and S1^17,20,36^. As can be seen by increasing the excitation wavelength from 310 to 350 nm, corresponding emissions intensities enhance accordingly (Fig. 4a), but by raising the excitation wavelength from 350 to 390 nm, emissions intensities decrease adversely (Fig. 4b). From these data it is deducted that strongest PL emission occurred at 437 nm when excited at 350 nm.

**Figure 5:**
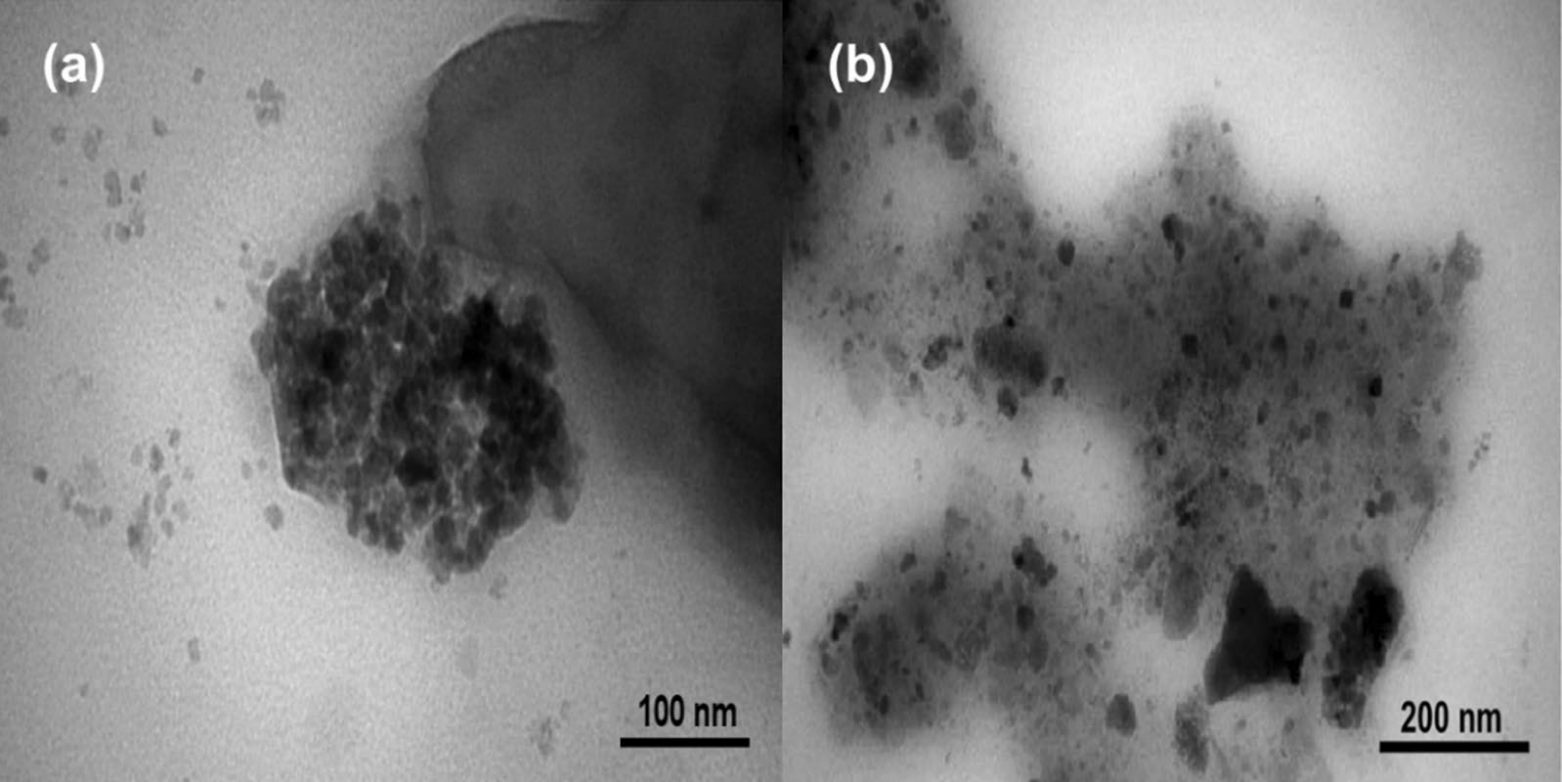
TEM images of A-CQDs/W, (**a**) 100 nm resolution, (**b**) 200 nm resolution.

In order to assess the shape morphology and distribution features of A-CQDs/W, TEM analysis was carried out (Figs. 5 and S2). As is shown, A-CQDs/W grains distributed evenly with no obvious accumulation with the sizes of approximately less than 10 nm.

**Figure 6:**
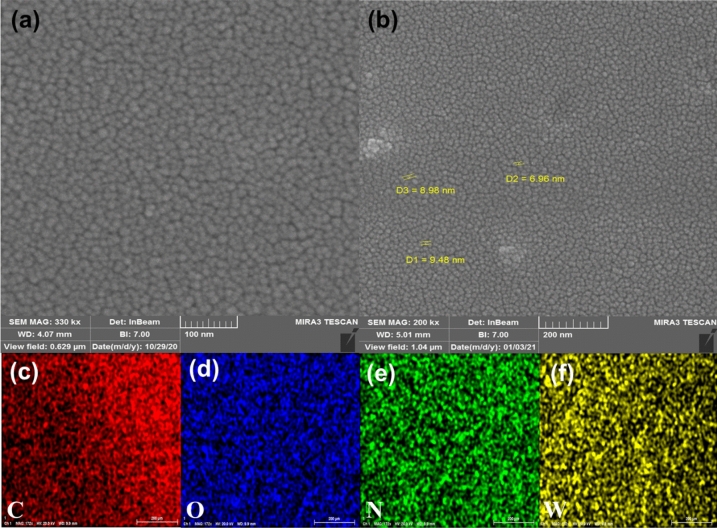
FESEM images of A-CQDs/W, (**a**) 100 nm resolution, (**b**) 200 nm resolution. Elemental mapping of A-CQDs/W, (**c**) Carbon, (**d**) Oxygen, (**e**) Nitrogen, (**f**) Tungsten.

FESEM imaging was carried out to study the surface morphology of A-CQDs/W sample (Figs. 6 and S3). As can be seen, catalyst particles are formed into fine, discrete and spherical grains with the mean size of about 10 nm. These images confirm the information deducted from TEM. The elemental structure of samples that are resulted from EDX mapping technique illustrate the uniform distribution of all C, O, N, and W atoms in catalyst and it can be seen that W atoms are evenly decorated throughout the CQDs surface which verifies successful [APMIm][Cl] loading and anion exchange on CQDs (Fig. 6c–f).

**Figure 7:**
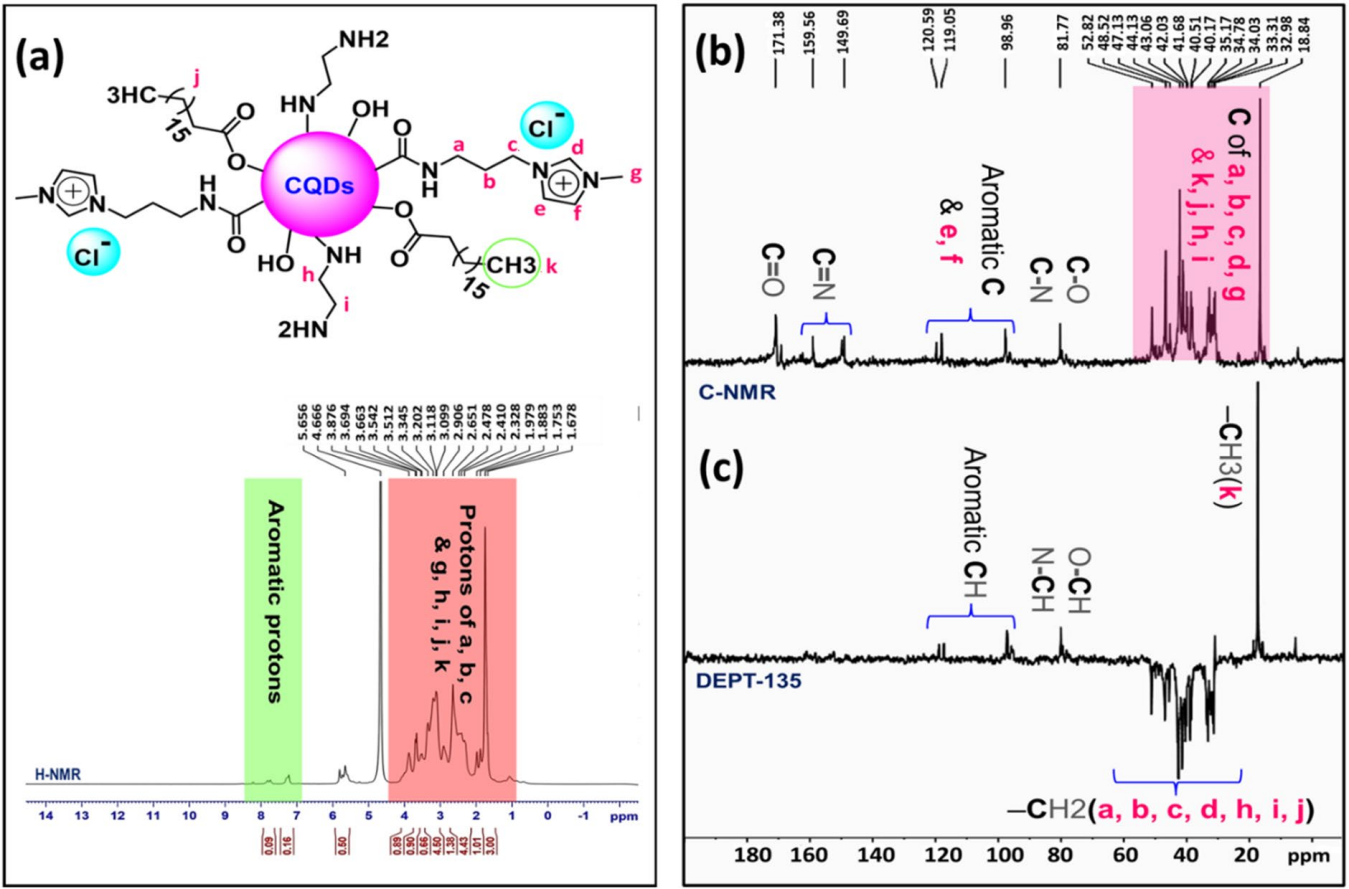
(**a**) ^1^H-NMR graph of A-CQDs**,** (**b**) ^13^C-NMR and (**c**) DEPT-135 NMR graph of A-CQDs.

NMR spectroscopy is a precious tool to identify the molecular structure and functional groups of compounds. ^1^H-NMR graph related to A-CQDs illustrated in Fig. 7a, as is shown, characteristic peak corresponds to saturated sp^3^ protons derived from [APMIm][Cl] and Stearic Acid, reflected at 1–3 ppm^34,37^. Meanwhile, peaks at 3–4.5 ppm are related to protons engaged with electronegative O or N atoms, such as C–OH and C-NH ones. In addition, aromatic protons and imidazolium moieties were visible at 7–8.5 ppm. Furthermore, based on the data from ^13^C NMR, (Fig. 7b), peaks related to sp^3^ carbon atoms originated from Stearic Acid, 1,2-Bis(3-aminopropylamino) ethane and [APMIm][Cl] could be observe at 20–58 ppm^23^. District peaks at 80–90 ppm assigned to sp^3^ C atoms linked to electron withdrawing specious. Sp^2^ carbons involved in imidazole and aromatic rings marked at 98–125 ppm. Signals between 148 and 161 ppm represent carbons involved in double bonds with N, such as ones that exist in imidazole rings. At the end, some peaks at 163–175 ppm are ascribed to carbonyl groups.

On the other hand, distortion less enhancement by polarization transfer (DEPT) analysis, could be so informative NMR method in distinguish between different carbon based on number of hydrogen atoms bonded to them (a CH_3_ group (methyl), a CH_2_ group (methylene), and a CH group (methine)). According to the DEPT 135 spectrum provided in Fig. 7c, positive peaks at 18.8 and 35.1 ppm could be assigned to CH_3_ of Stearic Acid and imidazole rings, respectively. The distinctly appearing negative peaks at 35.9–55.7 ppm reflect the signals related to CH_2_ groups ascribed to Stearic Acid, 1,2-Bis(3-aminopropylamino) ethane and [APMIm][Cl]. Furthermore, the CH-NH and CH-OH moieties were clearly distiguished through appearing positive peaks at 80–90 ppm. In addition, the aromatic CH groups caused appearing positive peaks in DEPT 135 analysis at 90–125 ppm^38,39^.

### Performance of catalyst in ultrasound-assisted alcohol oxidation

Because of high demand for aldehydes in industrial and pharmaceutical sectors, either as intermediate or final product, adapting an efficient process to produce them in high efficiency and selectivity is of great importance. Catalytic alcohol oxidation is one of the most common methods for synthesizing aldehydes^40^. Heterogeneous and homogeneous catalysts are two main types of catalysts that are used in this kind of conversions. Nevertheless, some downsides regarding applying these catalysts, such as low degree of interaction between catalyst and reactants ascribed to heterogeneous catalysts, low yield, using toxic organic solvents and introducing polluting PTC or surfactants, meanwhile, difficulties associated with recovery of homogeneous catalysts such as need for great amount of hazardous organic solvent or separating agents and energy have always been involving in using of these kinds of catalysts^41,42^. Thus, these downsides necessitate the need for thinking up a practical solution which beside the preserving plus points of these catalysts, ensure the least financial and environmental cost of oxidation reaction. In this regard, in our previous work we detailed an effort to design a pseudohomogeneous catalyst, that in addition to fulfilling the great interaction between reactants, eliminated the need for using toxic organic solvent or PTC^23^. In order to investigate catalyst capability, oxidizing potential of amphiphilic multifunctional catalyst was examined and we observed that A-CQDs/W were capable of oxidizing a wide range of alcoholic substances into analogue aldehydes with 100% selectivity and above the 95% yield in benign aqua media by using green H_2_O_2_ oxidant and without the need for adding PTC. Also, this catalyst afforded the big deal of efficient interaction between reactants in diphasic media and transformed high percent of substrates into products, consequently, amount of waste and unreacted reagents reduced remarkably. However, in spite of achieving high yield in this way, reaction still required high time and temperature to proceed, (2 h, 90 °C), which render it as a high time and energy consuming reaction**.** In the present study with the aim of overcoming the downsides related to our previous work, by utilizing ultrasonic technology as a substitution for thermal heating (reflux setup) to carry out oxidation reactions with the same catalyst. After conducting a set of experiments, we came across some great result in terms of selectivity and yield percent in far less time compared to previous work. This great result was achieved as a result of synergic effect between ultrasound irradiation and subtle design of catalyst. We choose benzyl alcohol as a testing model in presence of H_2_O_2_ and using H_2_O as solvent. In addition, we assessed the impact of various factors on sonocatalytic yield, namely, catalyst amount, oxidant ratio, types of solvent, types of additives, alcoholic substrate, reaction time and the power of ultrasound on reaction efficacy. First of all, sonocatalytic performance of sole Na_2_WO_4_ was examined. According to data listed in (Table 2, entry 1) sonocatalytic oxidation in presence of Na_2_WO_4_ resulted in trace amount of BeOH, which implies the key role of amphiphilic multifunctional CQDs in proceeding oxidation reaction as a biphasic interconnector. According to the fact that sodium tungstate just enables it to be dissolved in aqua phase and could not blend in organic phase, there would be no efficient interaction between active catalytic species and alcoholic phase in aqua media in normal condition to start oxidation reaction. After using 0.5 mol % of A-CQD/W catalyst in the same condition (Table 2, entry 3), we recorded 77% yield to benzaldehyde. By increasing the amount of catalyst from 0.5 to 1 mol % in constant condition (entry 4), yield percent experienced 21% increase and hit 93%. As is obvious, catalyst scale-up had a slight influence on productivity. After determining the optimal amount of catalyst, we performed some other reactions to investigate the role of oxidant ratio on yield. According to data summarized in Table 2, in the (2:1) molar ratio of oxidant in relation to substrate (entry 9), yield was 79%. By increasing the ratio of H_2_O_2_ from 2:1 to 3:1, we observed 18% increase in yield and recorded 93% of yield (entry 4), but this elevating yield percent didn’t maintain in further figures and after introducing ratio of 4:1, yield dropped to 67%, (entry 10). Thus, the best results obtained in 3:1 molar ratio (H_2_O_2_/substrate) with 93% yield, (entry 4). Similar trend in yield was observed in assessing the effect of oxidation time on the yield. By increasing the reaction time from 1 to 7 min, (entries 4,11,12 and13), yield percent decreased gradually. One justification for this loss could be the formation of acidic byproducts by prolonging the reaction time. In regard to the effect of ultrasound power on productivity, as it is shown, by increasing the ultrasound power from 60 to 80%, yield of benzaldehyde increased remarkably from 51 to 93%, (entries 4,6 and 7). In order to study the impact of various additives on oxidation yield we performed the reactions in presence of a number of various additives that are listed in Table 3^43^.

**Table 2:** The impacts of different reaction conditions on the sonooxidation of benzyl alcohol by A-CQDs/W.


Entry	Catalyst	Mol (%)	Time (min)	H_2_O_2_/Substrate (mmol/mmol)	Power (%)	Yield (%)^a^	Conversion (%)^a^
1	Na_2_WO_4_	1	5	3/1	80	Trace	Trace
2	A-CQDs	10 mg	5	3/1	80	13	16
3	A-CQDs/W	0.5	3	3/1	80	77	81
4	A-CQDs/W	1	3	3/1	80	93	98
5	A-CQDs/W	1.5	3	3/1	80	90	97
6	A-CQDs/W	1	3	3/1	70	75	80
7	A-CQDs/W	1	3	3/1	60	51	55
8	A-CQDs/W	1	3	0	80	Trace	Trace
9	A-CQDs/W	1	3	2/1	80	79	87
10	A-CQDs/W	1	3	4/1	80	67	71
11	A-CQDs/W	1	1	3/1	80	66	72
12	A-CQDs/W	1	5	3/1	80	86	70
13	A-CQDs/W	1	7	3/1	80	71	78

^a^Conversions and yields were calculated based on initial mmol of benzyl alcohol, (Isolated Yields).

**Table 3:** The impacts of different additives on the sonooxidation of benzyl alcohol by A-CQDs/W.


Entry	Additive	Yield (%)	Conversion (%)
1	KCl	92	95
2	KOH	71	74
3	Na_2_CO_3_	77	80
4	NaHCO_3_	80	83
5	H_2_SO_4_	94	96
6	NaHSO_4_	89	92

Optimal condition: Catalyst (1 mol%), Time (3 min), H_2_O_2_/Substrate (3 mmol/1 mmol), Power (80%). Conversions and yields were calculated based on initial mmol of benzyl alcohol, (Isolated Yields).

Different additives could induce the various pH values to reaction media which either could have a boosting or losing effect on reaction efficiency. All in all, in this work additives did not cause a dramatic change in yield, although slightly acidic media was in favor of oxidation reaction (Table 3, entry 5), but strong acidic or basic additives had an adverse effect on the reaction efficacy. As is shown in Fig. 9, in a light acidic media resulted from presence of carboxylic acid groups anchored on CQDs, bisperoxo tungstate that is resulted from the reaction between H_2_O_2_ and WO_4_^=^ tend to turn into more active mono protonated form (B species), which is highly likely to be fixed on A-CQDs and give (E species) the intermediate that later would be in charge of oxidation in organic phase. But in the intense acidic condition, inactive (D species) which are produced as a result of further protonation of (B species) are counted as the dominant species in aqua media and are unable to migrate to organic phase to trigger the oxidation reaction. Likewise, strong basic condition (entry 2) could result in formation of (C species) that causes a slump in yield. One reasonable explanation for this decrease could be the degradation of H_2_O_2_ in high pH values.

Regarding solvent influence on the reaction yield, we studied the effect of five different organic and inorganic solvents on the yield percent (Table 4). Amphiphilicity of catalyst enables it to perform well in aqua media without the need for using any kind of PTC or organic solvents, so, as the best performance of catalyst was recorded in H_2_O solvent (entry 1), we used water as green solvent in all the reactions in this work.

**Table 4:** The impacts of different solvents on the sonooxidation of benzyl alcohol by A-CQDs/W.


Entry	Solvent	Yield (%)	Conversion (%)
1	H_2_O	93	95
2	EtOH	73	76
3	DMF	71	74
4	DMSO	80	85
5	Acetonitrile	79	81
6	H_2_O/Acetonitrile	89	93
7	H_2_O/DMF	85	88

Optimal condition: Catalyst (1 mol%), Time (3 min), H_2_O_2_/Substrate (3 mmol/1 mmol), Power (80%). Conversions and yields were calculated based on initial mmol of benzyl alcohol, (Isolated Yields).

Beside the benzyl alcohol, the catalytic potential of A-CQDs/W in oxidizing some other alcoholic substrates was examined (Table 5). According to the results that are summed up in Table 5, benzyl alcohols substituted with electron withdrawing groups, (entry 3) resulted in higher yield towards the oxidation, compared to electron-donating substituent (entry 2), (95 vs 91%). Thus, substrates bearing electron-withdrawing groups are more productive reactants in sonocatalytic oxidation than pristine benzyl alcohol. Additionally, aromatic reactions afforded the better yield compared to aliphatic ones such as butanol and cyclohexanol with 84% and 88% yield respectively (entries 4 and 5).

**Table 5:** Sonooxidation of alcohols to corresponding aldehydes in the presence of A-CQDs/W catalyst.

Entry	Substrate	Product	Yield (%)	Conversion (%)
1			89	94
2			91	93
3			95	98
4			84	87
5			88	92

Optimal condition: Catalyst (1 mol%), Time (3 min), H_2_O_2_/Substrate (3 mmol/1 mmol), Power (80%). Conversions and yields were calculated based on initial mmol of benzyl alcohol, (Isolated Yields).

**Figure 8:**
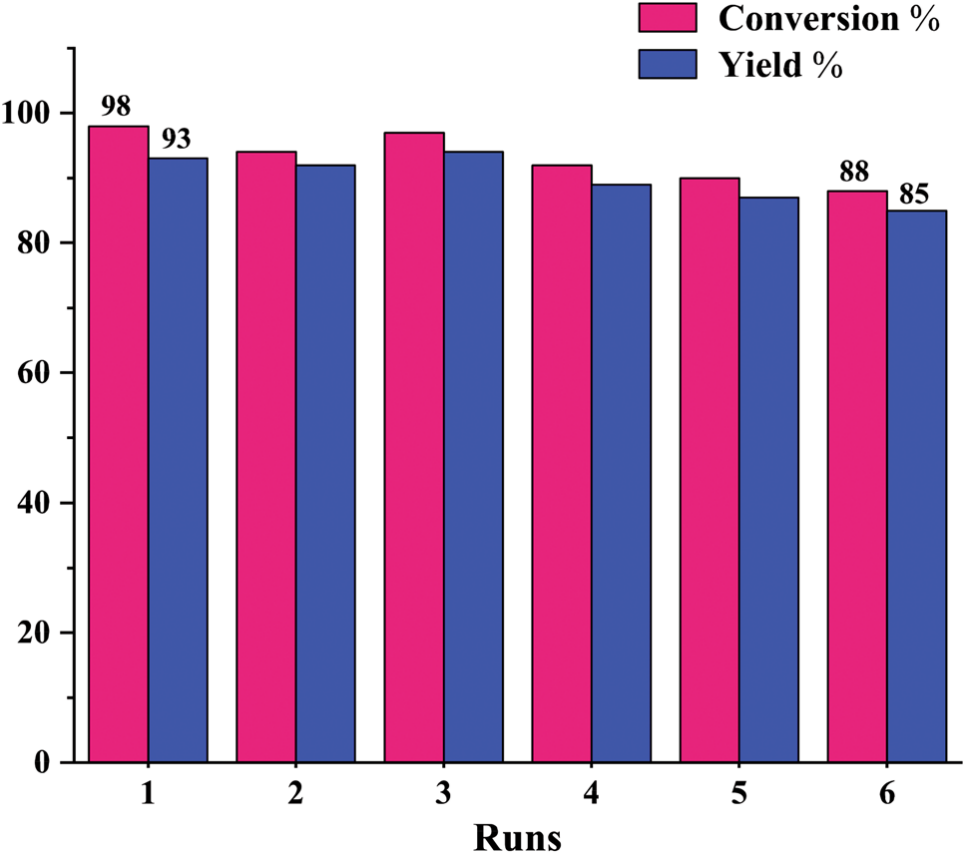
The reusability of A-CQDs/W in oxidation of benzyl alcohol.

At the end A-CQDs/W catalyst was easily separated by extraction. The vessel content was diluted by a mixture of water and ethyl acetate. Then, aqua phase containing the A-CQDs/W was isolated and dried under vacuum overnight and reused in 6 sequential runs without any obvious decline in catalyst efficiency which implies the superb performance and high stability of the green catalyst (Fig. 8).

### Proposed mechanism

**Figure 9:**
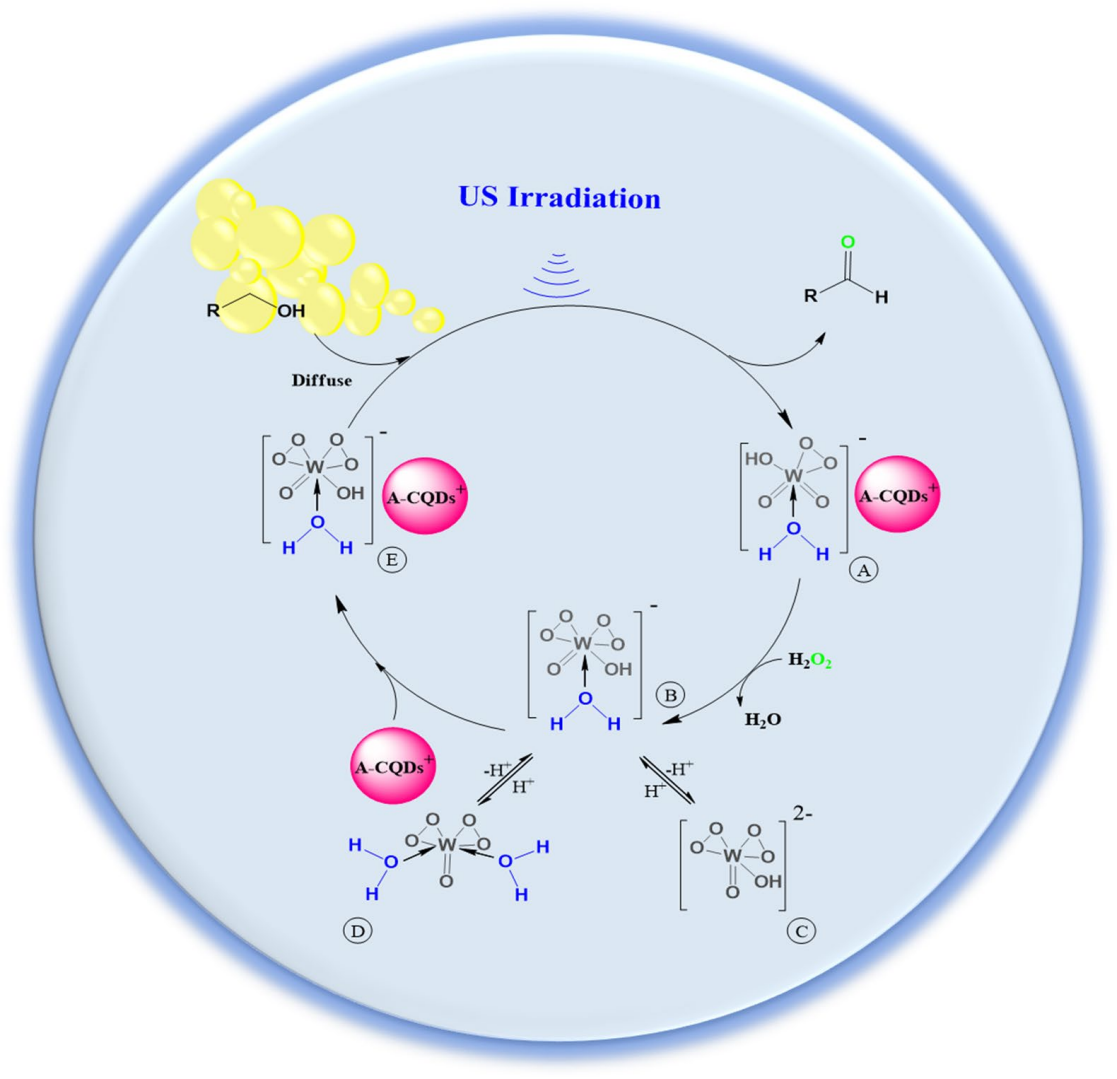
Schematic mechanism related to sonocatalitic oxidation of alcohol substrates into corresponding aldehydes.

According to the findings of current study and results reported in literature, we proposed a 4-step mechanism (Fig. 9)^20,22,23,44^. At the first and most important stage, as a result of reaction between H_2_O_2_ and A-CQDs/W_,_ all three (B, C) and (D) species could be generated in different proportions. However, the chance of being the dominant species in the vessel for each one of them highly depends on the degree of media acidity. In unadjusted pH condition after combining H_2_O_2_ with A-CQDs/W, slight acidic condition that is existed because of carboxylic acid groups anchored on A-CQDs, is in favor of producing bisperoxo tungstate, (B) species as the dominant compound in vessel. Bis peroxo tungstate is a critical for proceeding the oxidation, as this compound is in charge of oxidation of alcohol to corresponding aldehyde throughout the reaction. Division from favorable pH value, would result in dominance of (C, D) species that in section that impact of various additives on oxidation yield is discussed in detail. After this stage, an active (B) compound that is immobilized on A-CQDs via hydrophilic groups, would give the (E) intermediate. As a result of this substitution, active (B) agents would be capable of diffusing into organic alcoholic phase and with the assistance of ultrasound waves trigger the oxidation reaction. Finally, after placing the alcoholic ligand on A-CQDs/W and then ligand exchange reaction, aldehyde and (A) compound would be produce. It is worth mentioning that the precious feature of amphiphilicity in A-CQDs/W, not only enables it to smoothly spray in aqua phase but also promote the alcoholic species arrival into aqua phase through developing hydrogen bond between alcoholic part and polar sections of A-CQDs/W. It should be taken into consideration that high yield of products (73%) that is generated in presence of ethanol solvent (Table 4, entry 2) is in contrast with assumption of proceeding the sono oxidation reaction through radically route, so, the possibility of proceeding the reaction in this way would be rejected^28^.

## Conclusion

Multifunctional amphiphilic CQD base catalyst, A-CQDs/W, was synthesized through facile one pot hydrothermal method and used for selective oxidation of a range of alcoholic substrates into corresponding aldehydes by using H_2_O_2_ as oxidant and ultrasound effect as a green activation method. Applying ultrasound waves into reaction media proved to have a significant role in proceeding oxidation reactions and achieving high yield, increase in total greenness of reaction as well as remarkable decrease in energy consumption and reaction time. The resulting improvements stem from synergic effects between amphiphilic pseudohomogeneous catalysts and ultrasonic waves which caused enhanced mass transfer and unique reaction conditions in terms of pressure and temperature. By conducting this study, the immense potential of ultrasound waves in proceeding catalytic alcohol oxidation brought to the light that can inspire conducting further studies on ultrasound assisted transformations.

## Supplementary Information

Supplementary Information.
