# Supplementary material for: Ultrasound-assisted pseudohomogeneous tungstate catalyst for selective oxidation of alcohols to aldehydes
Source: Sci Rep. 2022 Mar 1;12:3367. doi: 10.1038/s41598-022-06874-5 (PMC8888602; doi:10.1038/s41598-022-06874-5)
Supplement: Supplementary file 1 — Supplementary Information. [file 41598_2022_6874_MOESM1_ESM.docx]

*Supporting Information*

**Ultrasound-Assisted Pseudohomogeneous Tungstate Catalyst for Selective Oxidation of Alcohols to Aldehydes**

Aram Rezaei,^¥*^ Yasaman Mohammadi,^€^ Ali Ramazani^€^ and Huajun Zheng^Δ*^

¥ Nano Drug Delivery Research Center, Health Technology Institute, Kermanshah University of Medical Sciences, Kermanshah, Iran.

€ Department of Chemistry, University of Zanjan, Zanjan, Iran.

Δ Department of Applied Chemistry, Zhejiang University of Technology, Hangzhou, 310032, China.

Corresponding Authers: Aram Rezaei, Email: [aram.rezaei@gmail.com](mailto:aram.rezaei@gmail.com); Huajun Zheng; Email: zhenghj@zjut.edu.cn.

Figure S1. PL graphs of A-CQDs sample, (a) Ex=310-350 nm, (b) Ex=350-390 nm


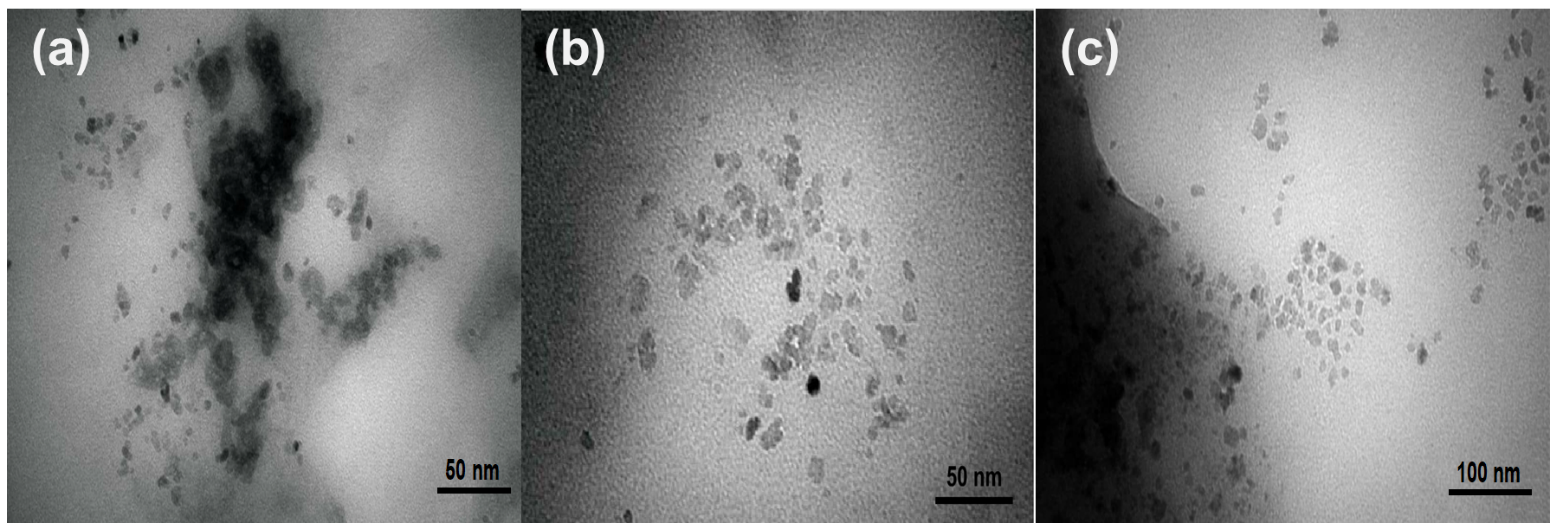


Figure S2. TEM image of the A-CQDs/W. (a) and (b) 50 nm resolution, (c) 100 nm resolution


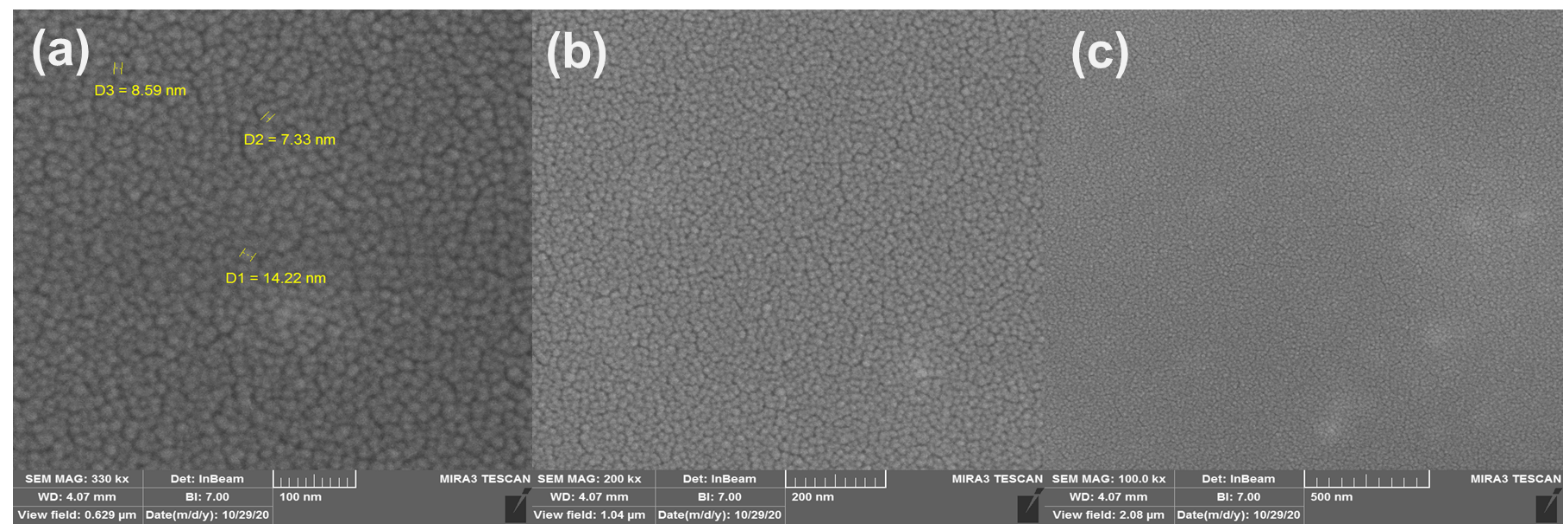


Figure S3. FE-SEM image of the A-CQDs/W. (a) 100 nm resolution, (b) 200 nm resolution, (c) 500 nm resolution
